# Glycerol-derived reuterin regulates human intestinal microbiota and metabolites

**DOI:** 10.3389/fmicb.2024.1454408

**Published:** 2024-10-18

**Authors:** Xi Yang, Wei Liu, Xiaoling Zhang, Minhua Sun, Hongbo Yi, Shenquan Liao, Rong Xiang, Hao Zhang, Qiao Yang, Hirotada Mori

**Affiliations:** ^1^State Key Laboratory of Livestock and Poultry Breeding, Guangdong Provincial Key Laboratory of Animal Breeding and Nutrition, Institute of Animal Science, Guangdong Academy of Agricultural Sciences, Guangzhou, China; ^2^Institute of Plant Protection and Microbiology, Zhejiang Academy of Agricultural Sciences, Hangzhou, China; ^3^ABI Group, College of Marine Science and Technology, Zhejiang Ocean University, Zhoushan, China; ^4^Key Laboratory of Livestock Disease Prevention of Guangdong Province, Maoming Branch of Guangdong Laboratory for Lingnan Modern Agriculture, Scientific Observation and Experiment Station of Veterinary Drugs and Diagnostic Techniques of Guangdong Province, Ministry of Agriculture and Rural Affairs, Institute of Animal Health, Guangdong Academy of Agricultural Sciences, Guangzhou, China; ^5^College of Pharmaceutical Sciences, Zhejiang Chinese Medical University, Hangzhou, China

**Keywords:** reuterin, antimicrobe, gut microbiota, intestinal gas production, SCFA

## Abstract

Reuterin, a mixture of different forms of 3-hydroxypropanal (3-HPA), including HPA hydrate and HPA dimer, is an antimicrobial compound converted from glycerol by *Lactobacillus reuteri and other strains*. Although its antimicrobial function may be related to its interaction with thiol groups, its temperature stability and effect on the gut environment remain unclear. The present study evaluated the antimicrobial effects and activity of reuterin against *Escherichia coli* and *Salmonella typhimurium*. Utilization of a reliable *in vitro* gut microbiome fermentation system revealed that reuterin has a modulatory effect on the gut microbial community. Reuterin treatment completely inhibited H_2_ and NH_3_ production in the gut and significantly enhanced the synthesis of branched short-chain fatty acids. 16s rRNA sequencing indicated that reuterin promoted the growth of Proteobacteria and Bacteroidetes in the *in vitro* system and significantly modulated gut microbiota composition.

## 1 Introduction

Antibiotic resistance is becoming a major health threat worldwide, making it important to find alternatives to traditional antibiotics that effectively suppress the growth of antibiotic-resistant pathogens (Zheng et al., 2020; Cleusix et al., 2008). *Lactobacillus reuteri* is commonly used as a probiotic micro-organism in food products, has been granted qualified presumption of safety (QPS) by the European Food Safety Authority (EFSA) (EFSA BIOHAZ Panel, 2012), as a metabolite of probiotics, reuterin is generally considered safe for the host. Moreover, reuterin is a water-soluble compound, its antibacterial activity is not affected by proteases or environmental PH (Engels et al., 2016). Reuterin is a broad-spectrum antimicrobial compound produced from glycerol by *Lactobacillus reuteri*, which was reassigned to the genus *Limosilactobacillus* in April 2020 (Zheng et al., 2020), a common lactic acid bacterium found in the human gastrointestinal tract, milk, and many other environments. Reuterin is a mixture of different forms of 3-hydroxypropanal (3-HPA), including HPA hydrate, and HPA dimer (Cleusix et al., 2008). Reuterin has been found to inhibit the growth of several common pathogens, including enterohemorrhagic *Escherichia coli* (EHEC), enterotoxigenic *E. coli* (ETEC), *Salmonella enterica*, *Shigella sonnei*, and *Vibrio cholerae* (Spinler et al., 2008). Recently, Bell et al. (2022) reported that reuterin exibited cytotoxicity inhibited colon cancer cell growth. But how reuterin modulate the gut microbiota and influence the host cell is still unclear. Owing to the antimicrobial activity of reuterin, it has been widely used in food production to control various food-borne pathogens (Langa et al., 2014; Arqués et al., 2015; Vimont et al., 2019). However, current review article also discussed the safety concern about employing reuterin to food industrial because the unrevealed antimicrobe mechanisms (Sun et al., 2022).

The gut microbiota play an indispensable role in maintaining the structure and function of the gastrointestinal tract, contributing to the metabolism of nutrients and drugs, the prevention of pathogen colonization, and gut barrier function (Bordalo Tonucci et al., 2017). The gut microbiota are closely associated with various human intestinal and metabolic diseases, including inflammatory bowel disease (IBD), irritable bowel syndrome (IBS), and diabetes (Bisgaard et al., 2011; Firrman et al., 2019). Microbiota in the colon efficiently metabolize proteins, breaking them down into smaller peptides and/or amino acids or converting them by fermentation into end products such as short-chain fatty acids (SCFAs) and gas (Bordalo Tonucci et al., 2017; Bisgaard et al., 2011; Firrman et al., 2019; Martín et al., 2005). An *in vitro* study showed that the byproducts of high protein fermentation correlate negatively with health and contribute to intestinal and metabolic diseases (Martín et al., 2005).

*Lactobacillus reuteri* is an anaerobic probiotic that exists in the host gastrointestinal tract and produces reuterin. It is a standard constituent of the flora in the human gut, especially in the stomach, small intestine, and colon (Bordalo Tonucci et al., 2017).

Reuterin is a stable compound that has been shown to inhibit the growth of bacteria. Reuterin may therefore be an alternative to antibiotics, especially in immunodeficient individuals, such as human infants (Martín et al., 2005) and HIV infected patients (Darmayanti et al., 2019). Although many studies have indicated that the antimicrobial activity of reuterin may be related to its interaction with thiol groups (Schaefer et al., 2010), its antibacterial mechanism of action and its effect on the composition of gut microbiota remain unclear.

The present study was designed to determine the antibacterial mechanism of action of reuterin on several bacterial species and its effect on the composition of the gut microbiota. A reliable system was established to measure the ability of reuterin to inhibit the growth of *E. coli* K-12 and *Salmonella typhimurium*. This study also analyzed the effects of reuterin on gas production and the synthesis of SCFAs, as well as its effects on the 16s rRNA profiles of bacteria and on the composition of human gut microbiota.

## 2 Materials and methods

### 2.1 Bacterial strains and culture media

*L. reuteri* ATCC55730 was used to produce reuterin, and *E. coli* K-12 and *S. typhimurium* LT2 were employed to investigate the antimicrobial effects of reuterin. *L. reuteri* ATCC55730 and *E. coli* K-12 are model organisms of *L. reuteri* and *E. coli*, respectively, both of the strains are maintained in our laboratory. *S. typhimurium* LT2 was purchased from Guangdong Microbial Culture Collection Center (GDMCC), with a preservation number of GDMCC 1.237.

*L. reuteri* ATCC55730 was inoculated into 2 mL De Man, Rogosa, and Sharpe (MRS) medium and cultured overnight at 37°C. One mL of this suspension was added to 100 mL fresh MRS medium and shaken at 150 rpm in a water bath shaker (Aohua THZ-H2A, Changzhou, China) overnight at 37°C overnight. Small amounts of frozen glycerol stocks of *E. coli* K-12 and *S. typhimurium* were scraped off and inoculated into 2 mL liquid Luria Broth medium, and cultured as described previously.

### 2.2 Production of reuterin

Reuterin was produced as previously described (Talarico et al., 1988). Briefly, 500 μL of *L. reuteri* ATCC 55730 preculture were inoculated into 50 mL MRS medium and shaken at 150 rpm for 3 h at 37°C. The 50 mL bacterial culture was transferred to 1 L of MRS broth containing 20 mM glycerol and cultured overnight at 37°C. Bacterial cells were harvested by centrifugation at 10,000 rpm at 4°C for 10 min, washed twice with 100 mM potassium phosphate buffer (pH 7.0), and resuspended in 300 mL 200 mM glycerol solution. The cells were subsequently incubated at 37°C for 3 h to induce reuterin biosynthesis. The cultures were centrifuged at 10,000 rpm for 10 min at 4°C, and the supernatants were filtered through 0.22 μm filters (Axygen, United States) to remove bacterial cells. This crude reuterin extract was stored at −80°C.

### 2.3 Quantification of reuterin

The concentration of reuterin was determined by colorimetry (Vollenweider et al., 2003), with acrolein standard curves constructed as described (Lüthi-Peng et al., 2002). Briefly, acrolein solutions were prepared at concentrations of 0.35 mM, 0.7 mM, 0.105 mM, 0.14 mM, 0.175 mM and 0.211 mM. Subsequently, 6 mL of each acrolein solution were mixed with 4.5 mL of tryptophan solution (0.01 M in 0.05 M HCl; stabilized with a few drops of toluene) and 18 mL hydrochloric acid (12 N), and incubated at 37°C for 20 min. The absorbance of each solution at 550 nm was measured and a standard curve was constructed. Reuterin concentration was estimated by comparing its absorbance at 550 nm with the standard curve.

Reuterin content was confirmed by GC-MS (Talarico and Dobrogosz, 1989). Briefly, lyophilized reuterin was derivatized by reaction with 1 mL N, O-bis(trimethylsilyl) trifluoroacetamide (BSTFA), followed by silanization at room temperature. Acetonitrile was added until the white precipitate completely dissolved. Each sample was sprayed with nitrogen and sealed in a vial for gas chromatography (GC)-MS, which was performed using an Agilent 7890A-5975C system. Briefly, a 1 μL aliquot of each reuterin derivative was injected into the GC at 280°C in split mode (50:1) with the helium carrier gas flow set to 1.1 mL/min. The samples were separated on a DB-5 capillary column (60 m × 0.25 mm, I.D., 0.25 μm Agilent), with the temperature set at 40°C for 3 min, followed by a 6°C per min ramp to 260°C. The transfer line and ion source temperatures were 200°C, and the mass range was from *m*/*z* 40 to 400.

### 2.4 Minimal inhibitory concentration measurement of *Escherichia coli* and *Salmonella typhimurium*

LB medium, containing serially diluted solutions of reuterin extract, at final concentrations of 0.1 mM, 0.2 mM, 0.3 mM, 0.4 mM, 0.5 mM, 0.6 mM, 0.75 mM, 1.0 mM, 1.25 mM, 1.5 mM, 1.75 mM, and 2.0 mM, were inoculated with 1% (v/v) of *E. coli* or *S. typhimurium* preculture and incubated overnight at 37°C. For growth curve measurements, the absorbance at 600 nm of the culture was measured every 30 min using a microplate reader (MD SpectraMax iD3, USA). LB medium containing 30 μg/mL of kanamycin was used as a negative control, as kanamycin has been shown to inhibit the growth of both *E. coli* and *S. typhimurium*, whereas LB medium without any antibiotics was used as a positive control.

### 2.5 Anaerobic fermentation of gut microbiota

Fecal samples were collected from 10 volunteers (five males and five females, aged 20 to 60 years). All the volunteers were in good physical condition, had no chronic diseases, and had not taken any prebiotics, antibiotics, or other drugs at least 3 months prior to sampling.

Five grams of fecal samples collected from each volunteer were added to 50 mL of 0.1 M PBS solution and vortexed for 10 s to a prepare 10% (w/v) fecal homogenate suspension. The suspension was filtered through a 300-mesh filter sieve (PAMPAS, China), and the supernatants containing bacteria were collected. These samples were diluted 10-fold into yeast extract, casitone and fatty acid (YCFA) medium, which contained tryptone 1% (w/v), 0.25% yeast extract (w/v), 0.1% cysteine (w/v), 0.2% heme (w/v), 20% vitamin (v/v), 0.045% KH_2_ PO_4_ (w/v), 0.045% K_2_HPO_4_ (w/v), 0.005% NaCl (w/v), 0.0064% CaCl_2·_2H_2_O (w/v), and 0.009% MgSO_4·_7H_2_O (w/v). Two types of YFCA medium were prepared, one without (Cont1) and the other with (Cont2) 200 mmol of glycerol. Samples were measured in YFCA medium containing low (LDR) and high (HDR) reuterin concentrations. The concentration of reuterin in the LDR group was the same as its minimal inhibitory concentration (MIC), whereas the concentration of reuterin in the HDR group was 10-fold higher than its MIC. The samples were anerobic cultured in fermentation vials for 24 h. A 1.5 mL aliquot of each tube was centrifuged at 10,000 rpm for 5 min, and the pellet was stored at −20°C until used.

### 2.6 *In vitro* production of gut gas

Gut gas diversity was determined using a fermentation gas analyzer system (Hangzhou Hailu Biotech, China). Fecal bacterial suspensions were transferred to fermentation vials with YCFA medium containing different concentrations of reuterin. Gas concentrations were measured by inserting a gas-impermeable syringe into the rubber cap of each fermentation vial. Carbon dioxide (CO_2_), hydrogen (H_2_)_,_ methane (CH_4_), hydrogen sulfide (H_2_S), and ammonia (NH_3_) were measured after 24 h of fermentation at 37°C.

### 2.7 GC-MS analysis of reuterin and *in vitro* produced SCFAs

The composition of SCFAs after fermentation was measured by gas chromatography (GC, GC − 2010 Plus, Shimadzu, Japan) with a DB-FFAP column (0.32 mm × 30 m × 0.5 μm, Agilent Technologies, United States), as described (Hu et al., 2013). The carrier gas was nitrogen, and the flow rate was set at 19.0 mL/min with a split ratio of 1:10. The temperature of both the detector and injection port was 250°C. Trans-2-butenoic acid was used as the internal standard (Bai et al., 2017).

### 2.8 16S rRNA sequencing analysis

The 16S rRNA sequences of fecal samples and bacterial precipitates were determined using the Illumina system. The amplification primers were F-terminal: 5′-ACTCCTACGGGAGCAGCAG-3′ (forward) and 5′-GGACTACHVGGGTWTCTAAT-3′ (reverse). Taxonomic analysis was performed using the UCLUST of QIIME (version 1.8.0) platform and was based on comparing operational taxonomic units (OTUs) with those in the SILVA database (Quast et al., 2013). Results showing at least 97% OTU similarity were collected for further analysis. The National Center for Biotechnology Information Short Read Archive received all human consensus sequencing data with accession no. PRJNA754838.

### 2.9 Statistical analysis

All statistical analyses were performed using GraphPad Prism 8 and R language (version: 4.0.8) software (R Core Team, 2013), with the figures produced using the package ggplot2 (Wickham, 2009). Results were reported as mean ± standard deviation (SD), and differences were analyzed by one-way analysis of variance (ANOVA) as described previously (Heiberger, 2015), followed by parametric *t*-tests.

## 3 Results

### 3.1 Production of reuterin and its components

The acrolein-tryptophan calibration curve showed an *R*^2^ > 0.99 with the equation *Y* = 0.6524*X* − 0.0001095, where *Y* was the absorbance at 550 nm and *X* was the sample concentration (Supplementary Figure S1). Thus, reuterin was produced at a concentration of 26.13 mM in a 200 mL crude reuterin extract.

**Figure 1:**
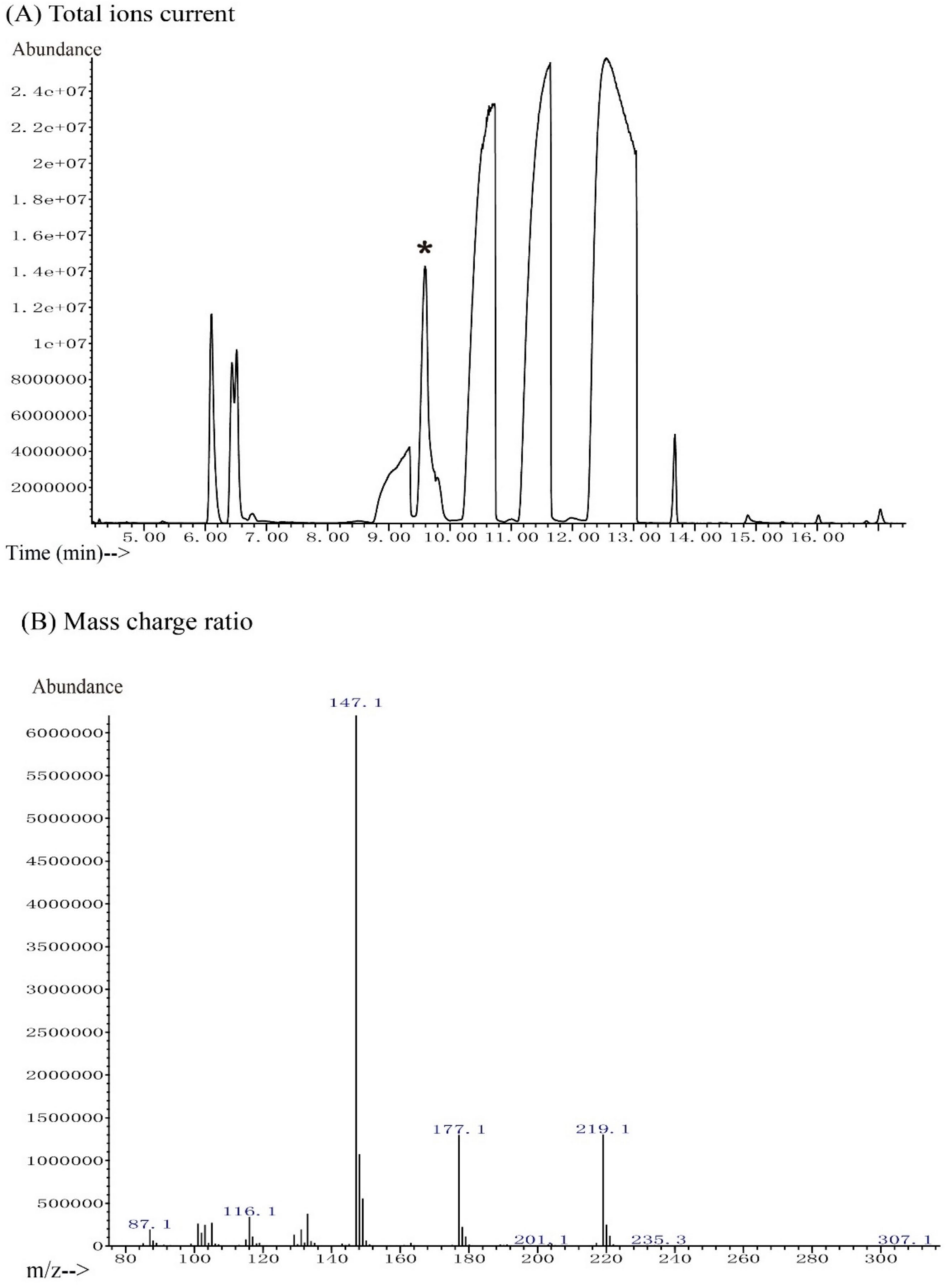
GC-MS chromatogram of reuterin. **(A)** The total ion current. The peak of reuterin derivatives was eluted from the GC at 9.59 min. The NIST database was employed for peak identification. The peak with an asterisk represents the 3-HPA peak. **(B)** Mass charge ratio. The peaks at *m*/*z* 147.1, 177.1, and 219.1 indicate 3-HPA in a stable isotope pattern.

Reuterin derivatives were produced after salinization with N, O-bis(trimethylsilyl)trifluoroacetamide (BSTFA) at room temperature. The derivatized sample showed a complex GC trace, in which a series of compounds were detected at retention times of 6 to 14 min (Figure 1A). The peak at 9.59 min was identified as a 3-hydroxypropionaldehyde dimer by screening the National Institute of Standards and Technology (NIST) chemical database (Figure 1B). The fragmentation pattern of this compound consisted of major signals at *m*/*z* 219.1, 177.1, and 147.1, which displayed a natural isotope pattern. These data confirmed that the molecule in this GC fraction was a cyclic dimer of 3-hydroxypropionaldehyde, consistent with previous findings (Talarico and Dobrogosz, 1989).

### 3.2 Antimicrobial activity

**Figure 2:**
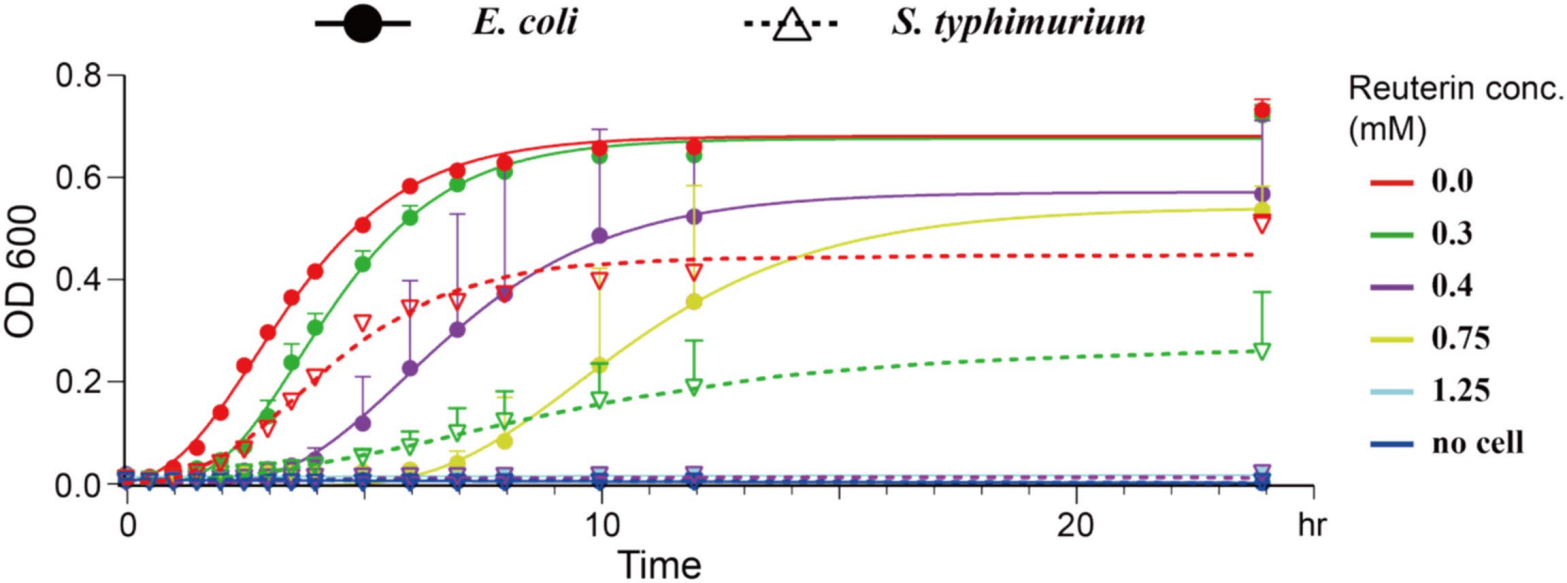
Reuterin inhibited bacterial growth. Solid circles represent *E. coli* K-12 growth, and empty inverted triangles represent *S. typhimurium* growth, as determined by absorbance at OD600, with the lines representing the fitted growth curves of *E. coli* K-12 and *S. typhimurium* at different concentrations of reuterin. The *X*-axis represents time points, and the *Y*-axis represents OD600 values; different colors represent different concentrations of reuterin, as shown at the top right. Growth was measured in bacteria culture in LB medium for 24 h at 37°C. The positive control indicates bacteria in LB medium without any drug, and the negative control indicates bacteria cultured in LB medium containing 30 μg/mL kanamycin. The curves were fitted using the Gompertz equation.

The antimicrobial activity of reuterin was determined by measuring the growth curve and MIC of *E. coli* K-12 and the human gut pathogen *S. typhimurium*. Although reuterin inhibited the growth of both *E. coli* K-12 and *S. typhimurium*, it was more effective against *S. typhimurium* under our experimental conditions (Figure 2). The minimal inhibition concentrations (MICs) of reuterin against *E. coli* K-12 and *S. typhimurium* were 1.25 mM and 0.4 mM, respectively.

Evaluation of reuterin temperature stability after storage for 24 h at −20°C, 25°C, and 37°C, and at 120°C for 20 min showed that Reuterin activity against both *E. coli* K-12 and *S. typhimurium* was very stable (Table 1).

**Table 1:** MICs of reuterin against *E. coli* K-12 and *S. typhimurium*.

	−20°C	25°C	37°C	120°C
*E. coli* K-12	1.25 ± 0.001	1.25 ± 0.001	1.25 ± 0.001	1.25 ± 0.184
*S. typhimurium*	0.4 ± 0.012	0.4 ± 0.023	0.4 ± 0.012	0.4 ± 0.130

Unit, mM. Each number represents the average of triplicate determinations.

### 3.3 Gas production during *in vitro* fermentation

**Figure 3:**
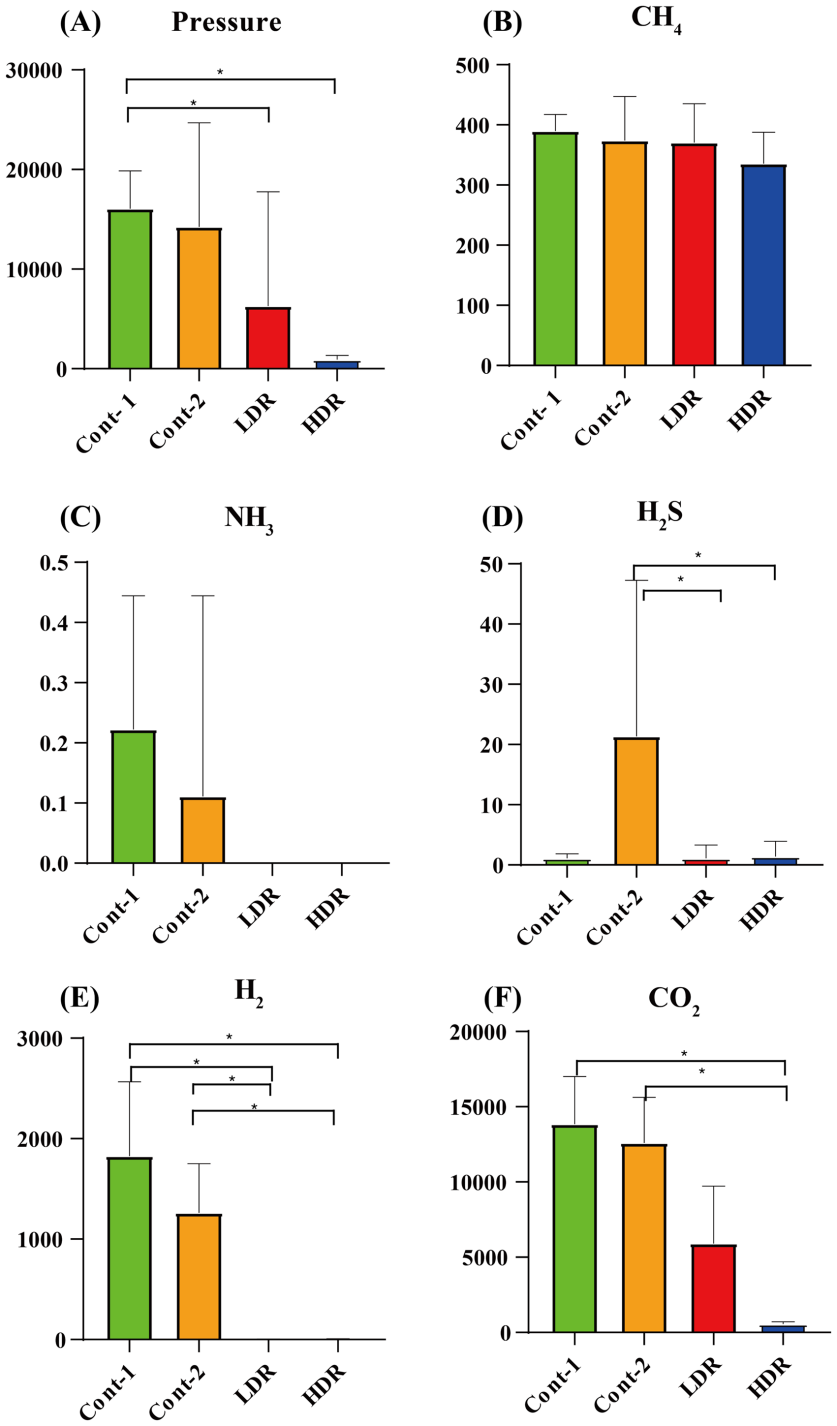
Production of gases by intestinal microbiota after *in vitro* fermentation for 24 h. **(A)** Average pressure in each experimental group. **(B–F)** Are the mean abundances of different types of gases in each group. Cont-1 consists of YCFA with gut sample, whereas Cont-2 consists of YCFA with glycerol and gut sample, LDR is the low-dose reuterin group, and HDR is the high-dose reuterin group. The *Y*-axis represents mean gas production, and the error bars represent the standard deviation. Each bar represents the mean ± SD of nine replicates, ^*^p-value <0.05, and ^**^p-value <0.01.

In this study, gas production and composition were investigated as indicators of fermentation rates. Reuterin was tested at low concentrations of 1.25 mM, the MIC of *E. coli* (LDR), and 12.5 mM, 10 times the MIC of *E. coli* (HDR). The mean value of each experimental group was determined, with one outlier that showed both abnormal gas measurement and SCFA detection (Supplementary Figures S2, S3) being excluded. Five types of fermentation-induced gases were detected, with the highest amount being carbon dioxide (CO_2_), followed by hydrogen (H_2_)_,_ methane (CH_4_), hydrogen sulfide (H_2_S), and a small amount of ammonia (NH_3_) (Figure 3). Compared with the Cont1 group, gas production correlated inversely with the concentration of reuterin extract (Figure 3A). Reuterin also showed different inhibitory effects on different types of gases. CH_4_ production in the HDR group decreased slightly, but there was no significant change in the LDR group (Figure 3B). H_2_S and CO_2_ production was not inhibited in the LDR and HDR groups (Figures 3D,F). H_2_ and NH_3_ production was completely inhibited in the HDR group (Figures 3C,E).

### 3.4 Reuterin interferes with SCFA metabolism *in vitro*

**Figure 4:**
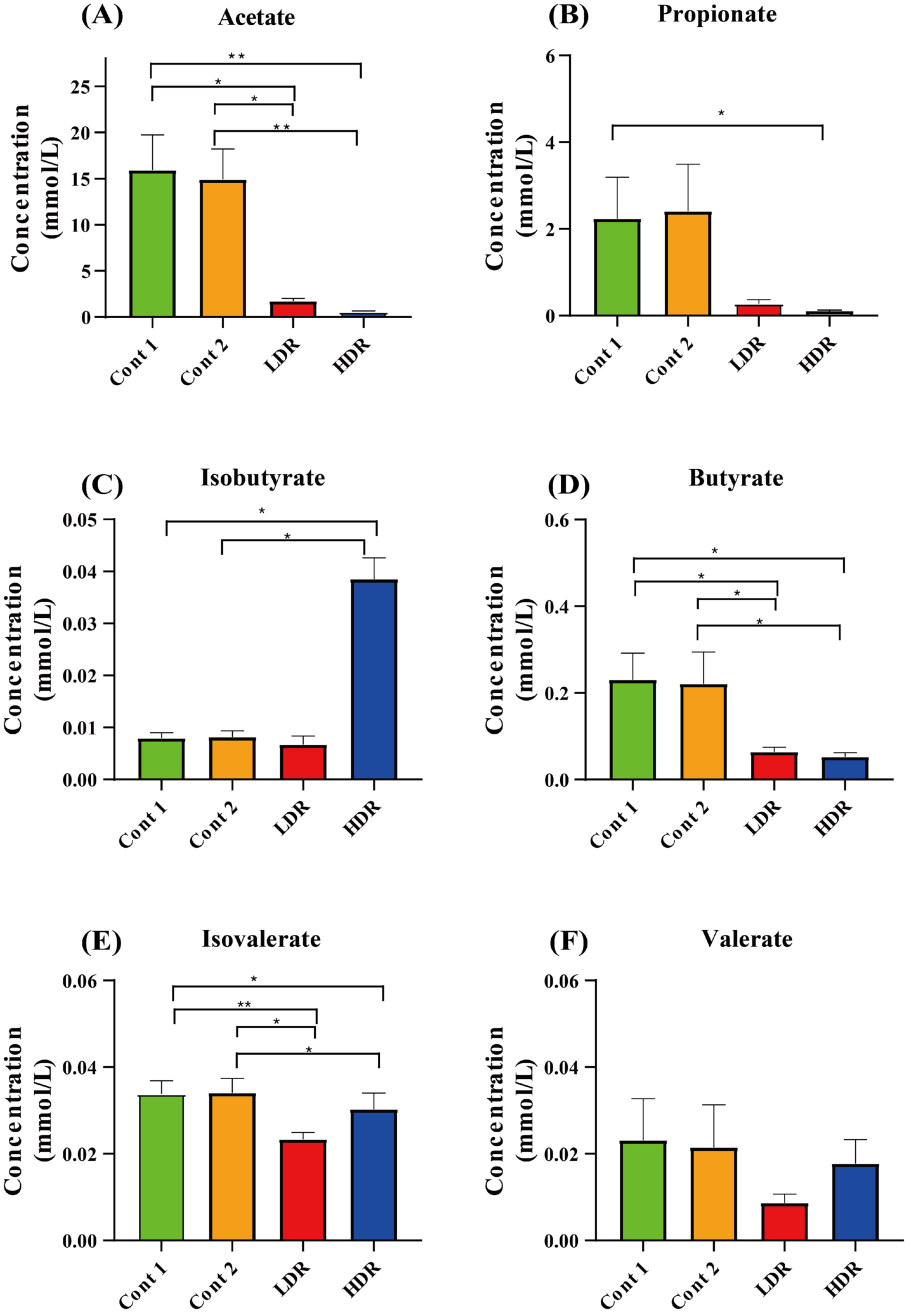
Production of SCFAs by intestinal microbiota after *in vitro* fermentation for 24 h. Cont-1 consists of YCFA with gut sample, whereas Cont-2 consists of YCFA with glycerol and gut sample, LDR is the low-dose reuterin group, and HDR is the high-dose reuterin group. The *Y*-axis represents mean SCFA concentration, and the error bars represent the standard deviation. Each bar represents the mean ± SD of nine replicates, ^*^p-value <0.05 and ^**^p-value <0.01.

Reuterin also reduced the production of most SCFAs in the *in vitro* fermentation system. The most abundant SCFA detected in the absence of reuterin was acetate, followed by propionate, butyrate, valerate, isobutyrate, and isovalerate (Figure 4). Reuterin significantly reduced the production of acetate, propionate, and butyrate (Figures 4A,B,D), while significantly increasing the production of isobutyrate (*p* < 0.05) (Figure 4C), compared with the control groups. The production of isovalerate and valerate was decreased in the LDR group but recovered in the HDR group. These results indicated that reuterin influenced isovalerate and valerate production in a dose-dependent manner (Figures 4E,F), while increases in the reuterin dose reduced the production of acetate, propionate, and butyrate (*p* < 0.05) (Figures 4A,B,D).

### 3.5 Reuterin influences the composition of gut microbiota *in vitro*

The changes in gut microbiota composition were investigated by 16s rRNA sequencing. Sequencing detected about 99.5% of the rDNA fragments with lengths of 401 to 500 bp from individual samples, showing that the data could be used to study variations in microbiota composition. A total of 1,772,929 valid sequences were analyzed from 40 samples, including two control groups (Cont-1 and Cont-2) and two test groups (LDR and HDR).

**Figure 5:**
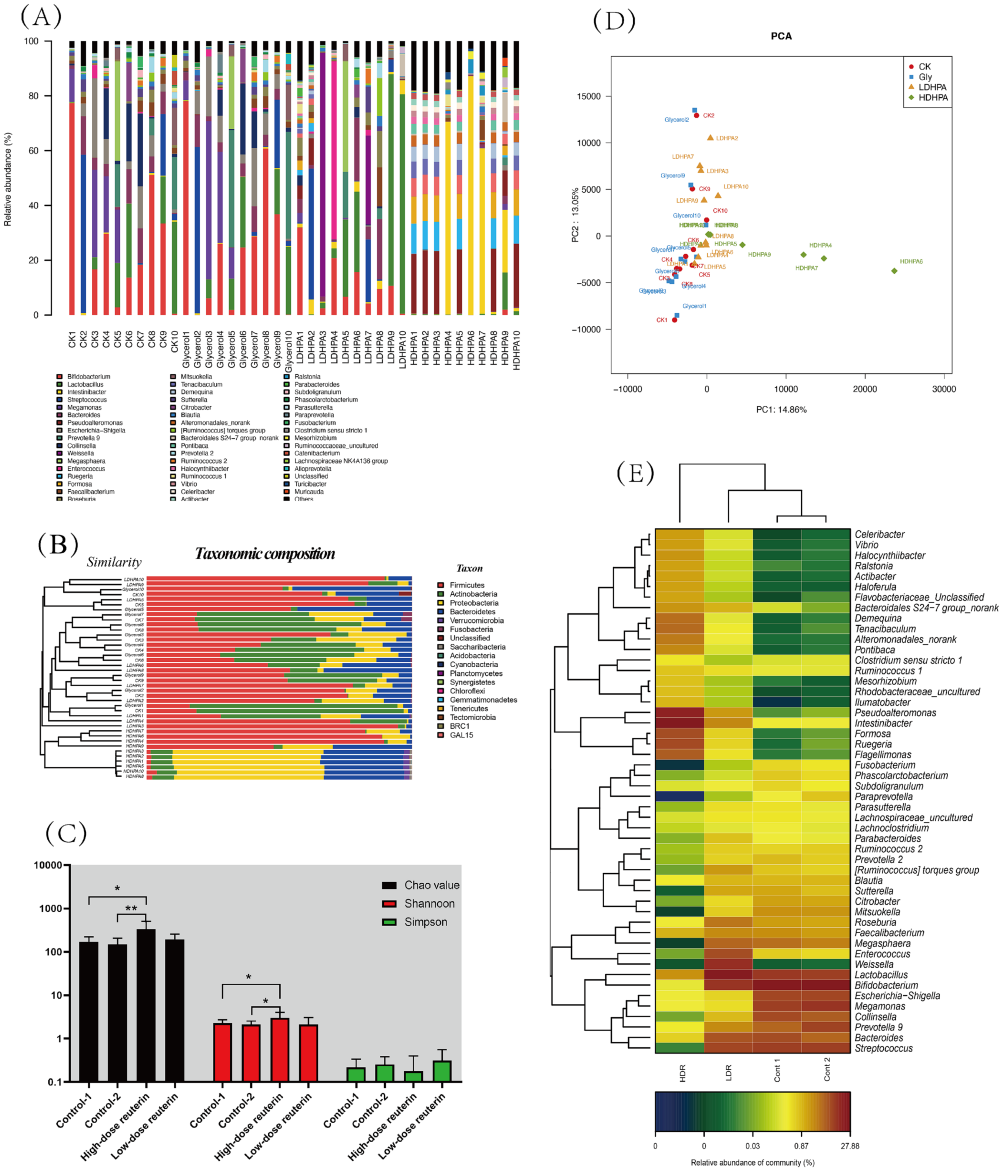
Reuterin influence gut microbiota composition and diversity. **(A)** Relative abundances of the bacterial community at the genus level. Each bar shows the distribution of enterobacterial species in each sample. The number on the *X*-axis represents the sample number. The *Y*-axis shows the relative abundance of bacterial distribution at the genus level in each sample as a percentage. The purple, green, red, and blue lines under each sample represent the sample groups for each culture condition: YCFA medium only (Cont-1), YCFA medium plus glycerol (Cont-2), and FCFA medium with low (LDR) and high (HDR) reuterin concentrations. **(B)** Microbial community barplot with cluster tree. The left side is the cluster of the bacterial composition base on Bray–Curtis method. The right side is taxonomic composition. **(C)** The alpha-diversity of microbiota communities. The black, red, and green bars represent the diversity values obtained using Chao, Shannon, and Simpson, respectively. Cont 1 consists of YCFA with gut sample. Cont 2 consists of YCFA with glycerol and gut sample. LDR is the low-dose reuterin group, and HDR is the high-dose reuterin group. ^*^p-value <0.05 and ^**^p-value <0.01. **(D)** Principal component analysis. Two axes represent two principal component, different color represents different sample groups, CK is the control group, Gly is glycerol fermentation group, LDHPA is the low dose reuterin sample group, HDHPA is the high dose reuterin sample group. **(E)** Correlation between reuterin and intestinal microbiota at the genus level. Each value represents the mean of 10 replicates. The *X*-axis represents sample groups, and the *Y*-axis represents bacteria at the genus level. Cont1 indicates YCFA medium with gut samples, Cont2 indicates YCFA medium with glycerol and gut samples, LDR indicates YCFA medium with low-dose reuterin and gut samples, and HDR indicates YCFA medium with high dose reuterin and gut samples. Colors represent the relative abundance of bacteria, increasing from blue to orange.

The evaluation of the gut microbiota distribution in each experimental group showed that HDR groups microbiota composition presents significant discrepancy and increased the microbiota diversity after *in vitro* fermentation for 24 h (Figure 5A; Supplementary Figures S4, S5), whereas the other groups showed no significant changes (*p* < 0.05). The dominant phyla in the microbiomes of the Cont-1, Cont-2, and LDR groups were *Firmicutes* and *Actinobacteria*, whereas the dominant phyla in the microbiomes of the HDR group were *Proteobacteria* and *Bacteroidetes* (Figure 5B). At the family level, diversity in the LDR group did not differ significantly from that in the control groups (Figure 5B; Supplementary Figures S4, S5) (*p* < 0.05), whereas *Pseudoalteromonadaceae*, *Flavobacteriaceae*, and *Rhodobacteraceae* ratios were significantly elevated in the HDR group (Supplementary Figure S5) (*p* < 0.05). At the genus level, *Bifidobacterium*, *Streptococcus*, *Megamonas*, and *Lactobacillus* were lower, whereas *Intestinibacter*, *Pseudoalteromonas*, *Ruegeria*, and *Mesorhizobium* were significantly higher (*p* < 0.05), in the HDR than in the control and LDR groups (Figure 5A).

**Figure 6:**
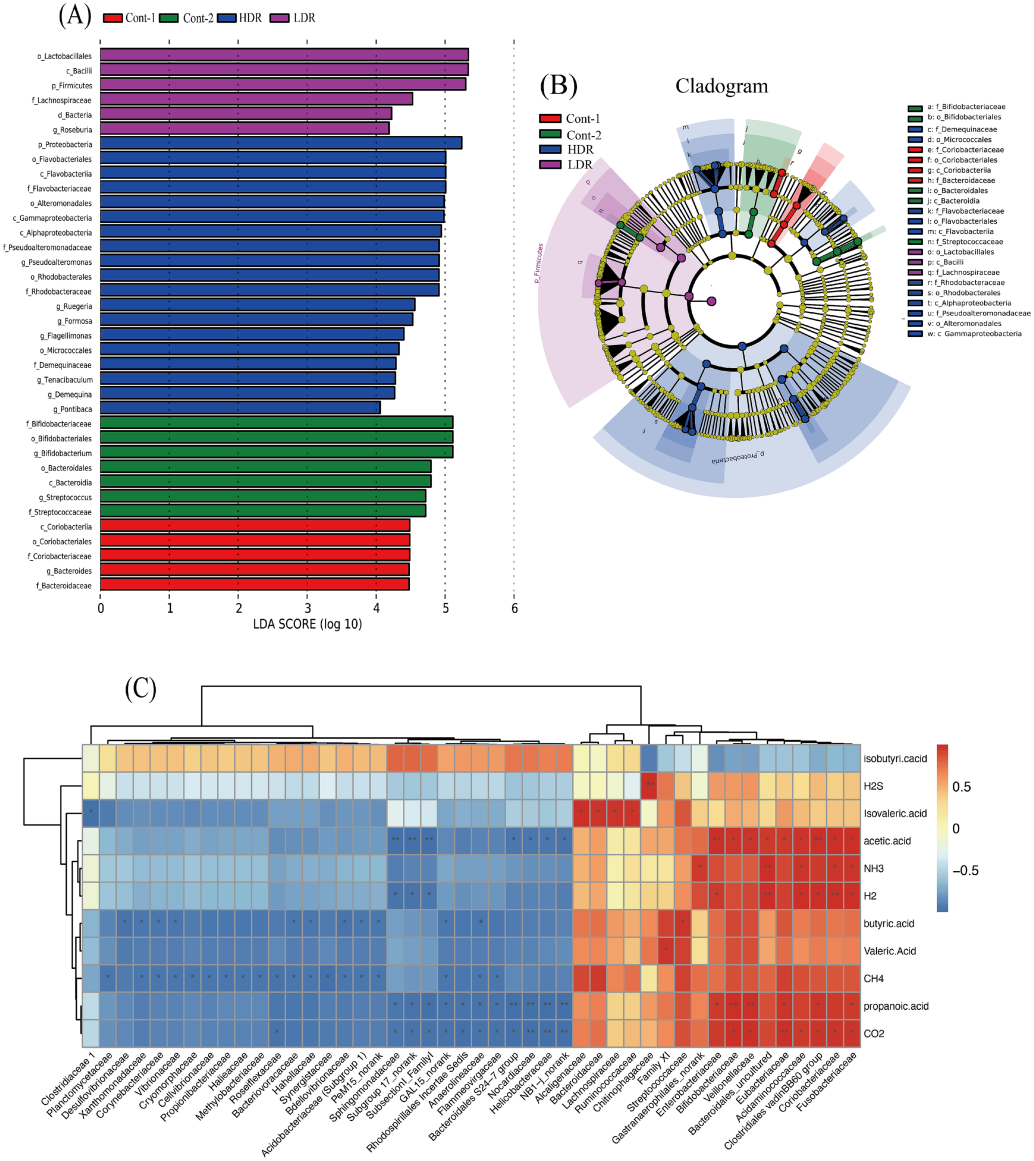
**(A)** LEfSe analysis of 24 h gut microbiota fermentation *in vitro*. The *X*-axis shows logarithmic LDA scores, and the *Y*-axis shows significant the names of diverse bacteria. LDA scores ≥2 with *p*-values <0.05 are indicative of significant diversity of species. **(B)** Cladogram of significantly diverse bacteria in each group. Different colors represent different sample groups, with nodes of the same color representing significantly diverse bacteria within the group and node sizes representing bacterial abundance. Red, green, blue and purple colors represent YCFA with gut sample (Cont-1), YCFA with glycerol and gut sample (Cont-2), high-dose reuterin group (HDR), and low-dose reuterin group (LDR), respectively. **(C)** Correlation between gas, metabolites and intestinal microbiota in family level. Each value represents the mean of 10 replicates. The *X*-axis represents bacterial family, and the *Y*-axis represents different gas or metabolites after reuterin fermentation. Colors represent the relative abundance of bacteria, increasing from blue to orange,“*” represents significance (*p*-values <0.05), “**” represents very significance (*p*-values <0.01).

To analyze the variation of microbial composition in each condition, the alpha-diversity of the gut microbiota composition was examined by comparing the Chao value, Shannon index, and Simpson index of each group. The findings suggested that reuterin might modulate the composition of the gut microbiota and therefore promote alpha-diversity of fecal microbiota. Both the Chao value and the Shannon index in the HDR group increased significantly (*p* < 0.01) (Figure 5C). Principle component analyis was performed, the results showed that gut microbiota of control systems, glycerol systems and LDR group samples was clustered together, whereas the bacterial composition of HDR group samples was more divers (Figure 5D). HDR significantly increased the relative amounts of *Pseudoalteromonas*, *Intestinibacter*, *Formosa*, *Ruegeria*, and *Flagellimonas* but reduced the relative amounts of *Streptococcus*, *Collinsella*, *Parasutterella*, and *Fusobacterium* at the genus level (*p* < 0.05) (Figure 5E). To further compare the diversity of the sample groups, Linear discriminant Effect Size (LEfSe) analysis was performed, in which the Linear discriminant analysis (LDA) score (Figure 6A) indicated the specific taxa that differed between sample groups. For example, *Bacteroidaceae* and *Coriobacteriaceae* were highly abundant in the Cont-1 groups, whereas the addition of glycerol resulted in *Bifidobacteriaceae* and *Streptococcaceae* becoming the most abundant families in the Cont-2 group. LDR increased the prevalence of *Lachnospiraceae*, whereas HDR significantly increased gut microbiome diversity (*p* < 0.05). *Demequinaceae*, *Rhodobacteraceae*, *Pseudoalteromonadaceae*, and *Flavobacteriaceae* dominated the gut microbiota community in the HDR group after 24 h of fermentation. Figure 6B shows the most diverse bacterial species at all levels. On family level, the *Demequinaceae*, *Flavobacteriaceae*, *Rhodobacteraceae*, and *Pseudoalteromonadaceae* have higher abundance in HDR group, and only *Lachnospiraceae* has higher abundance in LDR group (Figure 6B). Our data exhibited that reuterin influenced gas and SCFA synthesis in the gut (Figure 6C). H_2_S production had a very significant positive correlation with *Chitinophagaceae*, *p*-value <0.01. NH_3_ producing had very significant positive correllation with *Bacteroidales*, *p*-value <0.01 and significant positive correlation with *Gastranaerophilales*, *Acidaminococcaceae*, *Clostridiales vadin BB60 group* and *Fusobacteriaceae*, *p*-value <0.05. H_2_ production very significantly positively related to *Bacteroidales* and *Coriobacteriaceae*, *p*-value <0.01 and significant positive correlation with *Enterobacteriaceae*, *Acidaminococcaceae*, *Clostridiales vadin BB60 group* and *Fusobacteriaceae*, *p*-value <0.05; besides, H_2_ production had significant negative correlation with *Sphingomonadaceae*, *subgroup 17* and *Subsectionl*, *p*-value <0.05. CH_4_ production had significant negative correlation with *Planctomycetaceae*, *Xanthomonadaceae*, *Corynebacteriaceae*, *Vibrionaceae*, *Cryomorphaceae*, *Cellvibrionaceae*, *Propionibacteriaceae*, *Halieaceae*, *Methylobacteriaceae*, *Roseiflexaceae*, *Bacteriovoracaceae*, *Hahellaceae*, *Synergistaceae*, *Bdellovibrionaceae*, *Acidobacteriaceae*, and *PeM15*, *p*-value <0.05. CO_2_ production had very significant positive correlation with *Eubacteriaceae*, *p*-value <0.01, and very significant negative correlation with *Nocardiaceae*, *Helicobacteraceae*, and *NB1-j*, *p*-value <0.01. For the metabolites, the branched chain fatty acid (BCFA), isovaleric acid showed positive correlation to *Alcaligenaceae*, *Bacterioidaceae*, *Lachnospiraceae*, and *Ruminococcaceae*. and significant negative correlation with *Clostridiaceae*, *p*-value <0.05. Acetic acid presented very significant positive correlation with *Enterobacteriaceae* and *Clostridiales vadin BB60 group*, *p*-value <0.01 and very significant correlation with *Sphingomonadaceae*, *subgroup 17* and *Subsectionl*, *p*-value <0.01. Propanoic acid had very significant positive correlation with *Bifidobacteriaceae* and *Veilonellaceae*, *p*-value <0.01, meanwhile, it presented negative correlation with *Bacteroidales S24-7 group*, *Nocardiaceae*, *Helicobacteraceae*, and *NB1-J*.

## 4 Discussion

The application of reuterin has no clear standard now, in milk preservative, the reuterin concentration was chosen between 0.06–32.5 mM (Cleusix et al., 2007); in chicken breeding, up to 2 mM reuterin combined with lactic acid or microcin J25 were employed to get rid of salmonella spp. (Zhang et al., 2021); for ham preservation, a 16 mM purified reuterin combined with high hydrostatic pressure (HHP) was employed to extend the shelf life (Montiel et al., 2015). In 2022, Bell et al. (2022) demonstrated that reuterin suppresses colorectal cancer growth in the healthy gut microbiome through altering redox balance in a low concentration about 50 μM.

In this work, we detected two concentrations of reuterin. One is MIC value and an extreme over-dose value 10xMIC. The study evaluated the antimicrobial activity of reuterin against an experimental model prokaryote, *E. coli* K-12, and a common intestinal pathogen, *S. typhimurium*. *S. typhimurium* was more sensitive to reuterin than *E. coli*, although the mechanism underlying this difference was unclear. Long term storage temperature at −20°C, 25°C, and 37°C or short-term high-temperature treatment at 120°C for 20 min did not affect the antimicrobial activity of reuterin, suggesting that reuterin extract would be suitable for high-temperature granulation.

Reuterin, a metabolite of probiotics, also efficiently influenced the production of gut gas, the synthesis of SCFAs, and the composition of gut microbiota.

Reuterin reduced the pressure of intestinal gas, which may be related to the modulatory effects of reuterin on microbiota composition or its inhibitory effects on gas-producing bacteria. After fermentation, the production of CO_2_ significantly decreased (*p* < 0.05), and the production of H_2_ and NH_3_ was totally inhibited, in the reuterin-treated groups. CO_2_ is the major bacterial fermentation product formed from carbohydrates in the gut. It is excreted quickly via exhaled breath and is widely exploited in colonoscopy. H_2_ is an indicator of carbohydrate malabsorption or small intestinal bacterial overgrowth (Gasbarrini et al., 2009). Although the contribution of the H_2_ transformative process remains unclear, some microorganisms may utilize H_2_, such as reductive acetogens, which utilize H_2_ for acetate synthesis; methanogenic archaea, which transfer H_2_ to CH_4_; and sulfate-reducing bacteria, which transfer H_2_ to H_2_S (Nakamura et al., 2010). In the present study, CH_4_ production was not significantly altered by reuterin, whereas H_2_ and H_2_S production was significantly decreased. This may indicate that reuterin inhibited sulfate-reducing bacteria but did not influence methanogenic archaea. Ammonia (NH_3_) is the main factor causing hepatic encephalopathy, and downregulation of its production is frequently used to manage hepatic encephalopathy (Shawcross and Jalan, 2005).

SCFAs are monocarboxylic acids with a chain length of up to six carbons atoms. They are the most abundant products of bacterial anaerobic fermentation of dietary fibers in the intestine. Acetate (C2), propionate (C3), and butyrate (C4) are the most abundant SCFAs in the intestine (Markowiak-Kopeć and Śliżewska, 2020; Layden et al., 2013). Reuterin increased isobutyrate and isovalerate amounts, indicating that reuterin may promote branched SCFA (BCFA) synthesis. Carbohydrate glycolysis in the gut results in the production of butyrate and propionate, both of which have beneficial effects on intestinal epithelial cells (IECs) and immune cells by inducing intracellular or extracellular processes (Parada Venegas et al., 2019). Isobutyrate and isovalerate are involved in BCFA synthesis. BCFA levels have been associated with high protein diets (Aguirre et al., 2016; Pieper et al., 2012), with BCFA in the gut mainly derived from branched-chain amino acids in the genera *Bacteroides* and *Clostridium* (Aguirre et al., 2016). BCFA production is strongly affected by gut microbiota composition, which influences the availability of intestinal amino acids (Mack et al., 2016; Borghi et al., 2017). The increased BCFA level of the reuterin groups observed in the present study might alter the microbiome diversity of gut microbes, increasing the abundances of *Bacteroides* and *Clostridium* in the gut microbe population.

HDR was found to increase the diversity of gut microbiota, as shown by 16s rRNA sequencing after 24 h of *in vitro* fermentation. Analysis of bacterial composition at the phylum, family, and genus levels showed that reuterin increased the abundance of *Proteobacteria* and *Bacteroidetes* in the fecal fermentation system. Metagenomic sequencing showed that low gene counts were associated with a higher abundance of *Proteobacteria* and *Bacteroidetes* (Le Chatelier et al., 2013). Our study found that HDR did not strongly affect the number of 16s rRNA sequencing reads. Rather, the increased abundance of *Proteobacteria* and *Bacteroidetes* was not due to changes in gene counts but to as yet undetermined biological effect. According to the data, reuterin increased isovaleric acid secreted, it is closely related to the higher abundance of *Alcaligenaceae*, *Bacteroidaceae*, *Lachnospiraceae*, and *Ruminococcaceae* families (Figure 6C), meanwhile, isovaleric acid has the significant (*p*-value <0.05) negative correlation with *Clostridiaceae*, which is consists with the previous results that reuterin inhibit *Clostridiaceae* bacteria growth (Engevik et al., 2020; Gómez-Torres et al., 2014).

*Proteobacteria* is one of the most abundant phyla in the human gut microbiota. It is dominant phylum in the intestines of newborn mice, but its abundance decreases with age (Biagi et al., 2010). The dominance of *Proteobacteria* is related to the absence of B-cells and IgA, as both the lack of B-cells and the deletion of IgA encoding genes results in the persistent dominance of *Proteobacteria* in adult mice, along with persistent inflammation (Mirpuri et al., 2014). The *Bacteroidetes* generally constitute more than 50% of the gut microbiome (Qin et al., 2010; Human Microbiome Project Consortium, 2012). These bacteria mostly stay in the distal intestine and participate in fiber digestion and SCFA metabolism (McNeil, 1984). *Bacteriodetes* in the gut microbiome have stronger metabolic activity in obese than in lean individuals, although the abundance of *Bacteroidetes* is lower in obese persons (Kolmeder et al., 2015). *Proteobacteria* is a biomarker of many diseases, and their increased abundance is associated with increased inflammation (Rizzatti et al., 2017). The promotion of *Proteobacteria* by reuterin suggests that reuterin may act as an immune system stimulant, activating the immune system in the host gut. Because *Bacteriodetes* is considered a source of SCFAs in individuals consuming a high fiber diet (Johnson et al., 2017), the reuterin-associated increase in *Bacteriodetes* may boost the synthesis of SCFAs in the gut of these individuals. The differences in the sensitivity of gut bacteria to reuterin, as well as changes in the gut environment induced by reuterin, may therefore influence the diversity of intestinal microbes.

In summary, this study described the successful separation and purification of reuterin and demonstrated that it had antimicrobial activity against *E. coli* and *S. typhimurium*. The findings of the present study detected the influence of the new antimicrobe compound to human gut environment. It showed that, reuterin has a modulatory effect on the gut microbiota community, as well as effects on SCFA composition and gas production in the gut. While, additional studies are needed to fully understand the molecular mechanism by which reuterin modulates the gut environment. This could be achieved by screening for antimicrobe target molecules using the single-gene deletion library of the *E. coli* Keio collection (Baba and Mori, 2008), or the ASKA plasmid library (Kitagawa et al., 2005).

## Data Availability

The datasets presented in this study can be found in online repositories. The names of the repository/repositories and accession number(s) can be found at: https://www.ncbi.nlm.nih.gov/genbank/, 754838.

## Supplementary material

SUPPLEMENTARY FIGURE S1 Tryptophan acrolein standard curve. The *X*-axis is the concentration of acrolein, in mM, and the *Y*-axis is the OD550 value. Each point represents the mean ± SD of triplicate determinations.

SUPPLEMENTARY FIGURE S2 Gases produced by each candidate after fermentation for 24 h *in vitro*. The *X*-axis represents the order of candidates, and the *Y*-axis represents the corresponding amount of gas produced. The green, orange, red, and blue colors represent the Control 1, Control 2, low-dose reuterin, and high-dose reuterin groups, respectively.

SUPPLEMENTARY FIGURE S3 SCFAs produced by each candidate after fermentation for 24 h *in vitro*. The *X*-axis represents the order of candidates, and the *Y*-axis represents the relative amounts of SCFAs produced. The green, orange, red, and blue represent the Control 1, Control 2, low-dose reuterin, and high-dose reuterin groups, respectively.

SUPPLEMENTARY FIGURE S4 Absolute bacterial abundance at the family level in each candidate after fermentation for 24 h *in vitro*. The *X*-axis represents the order of candidates in each group. The left *Y*-axis represents the cluster of bacterial composition in each group, and the right *Y*-axis represents the family names of bacteria.

SUPPLEMENTARY FIGURE S5 Relative abundances of the bacterial community at the family level. Each bar shows the distribution of species in each sample. The number on the *X*-axis represents the sample number. The *Y*-axis shows the relative abundance of bacteria at the family level in each sample as a percentage. The purple, green, red, and blue lines under each sample represent the sample groups for each culture condition: YCFA medium only (Cont-1), YCFA medium plus glycerol (Cont-2), and FCFA medium with low (LDR) and high (HDR) reuterin concentrations.

## References

[ref1] Aguirre M. Eck A. Koenen M. E. Savelkoul P. H. M. Budding A. E. Venema K. (2016). Diet drives quick changes in the metabolic activity and composition of human gut microbiota in a validated *in vitro* gut model. Res. Microbiol. 167, 114–125. doi: 10.1016/j.resmic.2015.09.006, PMID: 26499094

[ref3] Arqués J. L. Rodríguez E. Langa S. Landete J. M. Medina M. (2015). Antimicrobial activity of lactic acid bacteria in dairy products and gut: effect on pathogens. Biomed. Res. Int. 2015:584183. doi: 10.1155/2015/584183, PMID: 25861634 PMC4378328

[ref4] Baba T. Mori H. (2008). The construction of systematic in-frame, single-gene knockout mutant collection in *Escherichia coli* K-12. Methods Mol. Biol. 2008, 171–181. doi: 10.1007/978-1-59745-321-9_11 18392967

[ref5] Bai S. Chen H. Zhu L. Liu W. Yu H. D. Wang X.. (2017). Comparative study on the *in vitro* effects of *Pseudomonas aeruginosa* and seaweed alginates on human gut microbiota. PLoS One 12:e0171576. doi: 10.1371/journal.pone.0171576, PMID: 28170428 PMC5295698

[ref6] Bell H. N. Rebernick R. J. Goyert J. Singhal R. Kuljanin M. Kerk S. A.. (2022). Reuterin in the healthy gut microbiome suppresses colorectal cancer growth through altering redox balance. Cancer Cell 40, 185–200.e6. doi: 10.1016/j.ccell.2021.12.001, PMID: 34951957 PMC8847337

[ref7] Biagi E. Nylund L. Candela M. Ostan R. Bucci L. Pini E.. (2010). Through ageing, and beyond: gut microbiota and inflammatory status in seniors and centenarians. PLoS One 5:e10667. doi: 10.1371/journal.pone.0010667, PMID: 20498852 PMC2871786

[ref8] Bisgaard H. Li N. Bonnelykke K. Chawes B. L. K. Skov T. Paludan-Müller G.. (2011). Reduced diversity of the intestinal microbiota during infancy is associated with increased risk of allergic disease at school age. J. Allergy Clin. Immunol. 128, 646–652.e5. doi: 10.1016/j.jaci.2011.04.060, PMID: 21782228

[ref9] Bordalo Tonucci L. Dos Santos K. M. De Luces Fortes Ferreira C. L. Ribeiro S. M. De Oliveira L. L. Martino H. S. (2017). Gut microbiota and probiotics: focus on diabetes mellitus. Crit. Rev. Food Sci. Nutr. 57, 2296–2309. doi: 10.1080/10408398.2014.934438 26499995

[ref10] Borghi E. Borgo F. Severgnini M. Savini M. N. Casiraghi M. C. Vignoli A. (2017). Rett syndrome: a focus on gut microbiota. Int. J. Mol. Sci. 18:344. doi: 10.3390/ijms18020344, PMID: 28178201 PMC5343879

[ref11] Cleusix V. Lacroix C. Vollenweider S. Duboux M. Le Blay G. (2007). Inhibitory activity spectrum of reuterin produced by *Lactobacillus reuteri* against intestinal bacteria. BMC Microbiol. 7:101. doi: 10.1186/1471-2180-7-101, PMID: 17997816 PMC2222629

[ref12] Cleusix V. Lacroix C. Vollenweider S. Le Blay G. (2008). Glycerol induces reuterin production and decreases *Escherichia coli* population in an *in vitro* model of colonic fermentation with immobilized human feces. FEMS Microbiol. Ecol. 63, 56–64. doi: 10.1111/j.1574-6941.2007.00412.x, PMID: 18028400

[ref13] Darmayanti A. T. Susilawati T. N. Murti B. (2019). Giving probiotic for a better therapy of bacterial vaginosis. KnE Life Sci. 4, 239–246. doi: 10.18502/kls.v4i12.4179

[ref14] EFSA BIOHAZ Panel (2012). Scientific opinion on the development of a risk ranking framework on biological hazards. EFSA J. 10:2724. doi: 10.2903/j.efsa.2012.2724

[ref15] Engels C. Schwab C. Zhang J. Stevens M. J. A. Bieri C. Ebert M. O.. (2016). Acrolein contributes strongly to antimicrobial and heterocyclic amine transformation activities of reuterin. Sci. Rep. 6:36246. doi: 10.1038/srep36246, PMID: 27819285 PMC5098142

[ref16] Engevik M. A. Danhof H. A. Shrestha R. Chang-Graham A. L. Hyser J. M. Haag A. M.. (2020). Reuterin disrupts *Clostridioides difficile* metabolism and pathogenicity through reactive oxygen species generation. Gut Microbes 12:1795388. doi: 10.1080/19490976.2020.1795388, PMID: 32804011 PMC7524292

[ref17] Firrman J. Liu L. Tanes C. Friedman E. S. Bittinger K. Daniel S.. (2019). Metabolic analysis of regionally distinct gut microbial communities using an *in vitro* platform. J. Agric. Food Chem. 68, 13056–13067. doi: 10.1021/acs.jafc.9b05202, PMID: 31690071

[ref18] Gasbarrini A. Corazza G. R. Gasbarrini G. Montalto M. Di Stefano M. Basilisco G.. (2009). Methodology and indications of H2-breath testing in gastrointestinal diseases: the Rome Consensus Conference. Aliment. Pharmacol. Ther. 29, 1–49. doi: 10.1111/j.1365-2036.2009.03951.x, PMID: 19344474

[ref19] Gómez-Torres N. Ávila M. Gaya P. Garde S. (2014). Prevention of late blowing defect by reuterin produced in cheese by a *Lactobacillus reuteri* adjunct. Food Microbiol. 42, 82–88. doi: 10.1016/j.fm.2014.02.018, PMID: 24929721

[ref2] Heiberger R. M. Holland B. (2015). One-way analysis of variance. In: Statistical analysis and data display. Springer texts in statistics. New York, NY: Springer.

[ref20] Hu J.-L. Nie S.-P. Li C. Xie M.-Y. (2013). *In vitro* fermentation of polysaccharide from the seeds of *Plantago asiatica* L. by human fecal microbiota. Food Hydrocoll. 33, 384–392. doi: 10.1016/j.foodhyd.2013.04.006

[ref21] Human Microbiome Project Consortium (2012). Structure, function and diversity of the healthy human microbiome. Nature 486, 207–214. doi: 10.1038/nature11234 22699609 PMC3564958

[ref22] Johnson E. L. Heaver S. L. Walters W. A. Ley R. E. (2017). Microbiome and metabolic disease: revisiting the bacterial phylum Bacteroidetes. J. Mol. Med. 95, 1–8. doi: 10.1007/s00109-016-1492-2, PMID: 27900395 PMC5187364

[ref23] Kitagawa M. Ara T. Nakamichi T. Inamoto E. Toyonaga H. Mori H. (2005). Complete set of ORF clones of *Escherichia coli*: unique resources for biological research. DNA Res. 12, 291–299. doi: 10.1093/dnares/dsi012, PMID: 16769691

[ref24] Kolmeder C. A. Ritari J. Verdam F. J. Muth T. Keskitalo S. Varjosalo M.. (2015). Colonic metaproteomic signatures of active bacteria and the host in obesity. Proteomics 15, 3544–3552. doi: 10.1002/pmic.201500049, PMID: 26255997

[ref25] Langa S. Martín-Cabrejas I. Montiel R. Landete J. M. Medina M. Arqués J. L. (2014). Combined antimicrobial activity of reuterin and diacetyl against foodborne pathogens. J. Dairy Sci. 97, 6116–6121. doi: 10.3168/jds.2014-8306, PMID: 25087026

[ref26] Layden B. T. Angueira A. R. Brodsky M. Durai V. Lowe W. L. (2013). Short chain fatty acids and their receptors: new metabolic targets. Transl. Res., 161, 131–140. doi: 10.1016/j.trsl.2012.10.007 23146568

[ref27] Le Chatelier E. Nielsen T. Qin J. Prifti E. Hildebrand F. Falony G.. (2013). Richness of human gut microbiome correlates with metabolic markers. Nature 500, 541–546. doi: 10.1038/nature12506, PMID: 23985870

[ref28] Lüthi-Peng Q. Dileme F. Puhan Z. (2002). Effect of glucose on glycerol bioconversion by *Lactobacillus reuteri*. Appl. Microbiol. Biotechnol. 59, 289–296. doi: 10.1007/s00253-002-1002-z 12111160

[ref29] Mack I. Cuntz U. Grämer C. Niedermaier S. Pohl C. Schwiertz A.. (2016). Weight gain in anorexia nervosa does not ameliorate the faecal microbiota, branched chain fatty acid profiles and gastrointestinal complaints. Sci. Rep. 6:26752. doi: 10.1038/srep26752 27229737 PMC4882621

[ref30] Markowiak-Kopeć P. Śliżewska K. (2020). The effect of probiotics on the production of short-chain fatty acids by human intestinal microbiome. Nutrients 12:1107. doi: 10.3390/nu12041107, PMID: 32316181 PMC7230973

[ref31] Martín R. Olivares M. Marín M. L. Xaus J. Fernández L. Rodríguez J. M. (2005). Characterization of a reuterin-producing *Lactobacillus coryniformis* strain isolated from a goat’s milk cheese. Int. J. Food Microbiol. 104, 267–277. doi: 10.1016/j.ijfoodmicro.2005.03.007, PMID: 15975679

[ref32] McNeil N. I. (1984). The contribution of the large intestine to energy supplies in man. Am. J. Clin. Nutr. 39, 338–342. doi: 10.1093/ajcn/39.2.338, PMID: 6320630

[ref33] Mirpuri J. Raetz M. Sturge C. R. Wilhelm C. L. Benson A. Savani R. C.. (2014). Proteobacteria-specific IgA regulates maturation of the intestinal microbiota. Gut Microbes 5, 28–39. doi: 10.4161/gmic.26489, PMID: 24637807 PMC4049932

[ref34] Montiel R. Martín-Cabrejas I. Medina M. (2015). Reuterin, lactoperoxidase, lactoferrin and high hydrostatic pressure on the inactivation of food-borne pathogens in cooked ham. Food Control 51, 122–128. doi: 10.1016/j.foodcont.2014.11.010

[ref35] Nakamura N. Lin H. C. McSweeney C. S. Mackie R. I. Gaskins H. R. (2010). Mechanisms of microbial hydrogen disposal in the human colon and implications for health and disease. Annu. Rev. Food Sci. Technol. 1, 363–395. doi: 10.1146/annurev.food.102308.124101, PMID: 22129341

[ref36] Parada Venegas D. De la Fuente M. K. Landskron G. González M. J. Quera R. Dijkstra G.. (2019). Short chain fatty acids (SCFAs)-mediated gut epithelial and immune regulation and its relevance for inflammatory bowel diseases. Front. Immunol. 10:277. doi: 10.3389/fimmu.2019.00277, PMID: 30915065 PMC6421268

[ref37] Pieper R. Kröger S. Richter J. F. Wang J. Martin L. Bindelle J.. (2012). Fermentable fiber ameliorates fermentable protein-induced changes in microbial ecology, but not the mucosal response, in the colon of piglets. J. Nutr. 142, 661–667. doi: 10.3945/jn.111.156190, PMID: 22357743

[ref38] Qin J. Li R. Raes J. Arumugam M. Burgdorf K. S. Manichanh C.. (2010). A human gut microbial gene catalogue established by metagenomic sequencing. Nature 464, 59–65. doi: 10.1038/nature08821, PMID: 20203603 PMC3779803

[ref39] Quast C. Pruesse E. Yilmaz P. Gerken J. Schweer T. Yarza P.. (2013). The SILVA ribosomal RNA gene database project: improved data processing and web-based tools. Nucleic Acids Res. 41, D590–D596. doi: 10.1093/nar/gks1219, PMID: 23193283 PMC3531112

[ref40] R Core Team. (2013). R: A language and environment for statistical computing

[ref41] Rizzatti G. Lopetuso L. R. Gibiino G. Binda C. Gasbarrini A. (2017). Proteobacteria: a common factor in human diseases. Biomed. Res. Int. 2017:9351507. doi: 10.1155/2017/9351507, PMID: 29230419 PMC5688358

[ref42] Schaefer L. Auchtung T. A. Hermans K. E. Whitehead D. Borhan B. Britton R. A. (2010). The antimicrobial compound reuterin (3-hydroxypropionaldehyde) induces oxidative stress via interaction with thiol groups. Microbiology 156, 1589–1599. doi: 10.1099/mic.0.035642-0, PMID: 20150236 PMC7336520

[ref43] Shawcross D. Jalan R. (2005). The pathophysiologic basis of hepatic encephalopathy: central role for ammonia and inflammation. Cell. Mol. Life Sci. 62, 2295–2304. doi: 10.1007/s00018-005-5089-0 16158192 PMC11139067

[ref44] Spinler J. K. Taweechotipatr M. Rognerud C. L. Ou C. N. Tumwasorn S. Versalovic J. (2008). Human-derived probiotic *Lactobacillus reuteri* demonstrate antimicrobial activities targeting diverse enteric bacterial pathogens. Anaerobe 14, 166–171. doi: 10.1016/j.anaerobe.2008.02.001, PMID: 18396068 PMC2494590

[ref45] Sun M. C. Hu Z. Y. Li D. D. Chen Y. X. Xi J. H. Zhao C. H. (2022). Application of the reuterin system as food preservative or health-promoting agent: a critical review. Foods 11:4000. doi: 10.3390/foods11244000, PMID: 36553742 PMC9778575

[ref46] Talarico T. L. Casas I. A. Chung T. C. Dobrogosz W. J. (1988). Production and isolation of reuterin, a growth inhibitor produced by *Lactobacillus reuteri*. Antimicrob. Agents Chemother. 32, 1854–1858. doi: 10.1128/AAC.32.12.1854, PMID: 3245697 PMC176032

[ref47] Talarico T. L. Dobrogosz W. J. (1989). Chemical characterization of an antimicrobial substance produced by *Lactobacillus reuteri*. Antimicrob. Agents Chemother. 33, 674–679. doi: 10.1128/AAC.33.5.674, PMID: 2751282 PMC172512

[ref48] Vimont A. Fernandez B. Ahmed G. Fortin H.-P. Fliss I. (2019). Quantitative antifungal activity of Reuterin against food isolates of yeasts and moulds and its potential application in yogurt. Int. J. Food Microbiol. 289, 182–188. doi: 10.1016/j.ijfoodmicro.2018.09.005, PMID: 30253311

[ref49] Vollenweider S. Grassi G. König I. Puhan Z. (2003). Purification and structural characterization of 3-hydroxypropionaldehyde and its derivatives. J. Agric. Food Chem. 51, 3287–3293. doi: 10.1021/jf021086d 12744656

[ref50] Wickham H. (2009). Ggplot2: elegant graphics for data analysis. 2nd Edn. New York: Springer.

[ref51] Zhang L. Ben Said L. Diarra M. S. Fliss I. (2021). Inhibitory activity of natural synergetic antimicrobial consortia against *Salmonella enterica* on broiler chicken carcasses. Front. Microbiol. 12:656956. doi: 10.3389/fmicb.2021.656956, PMID: 33995320 PMC8116713

[ref52] Zheng J. Wittouck S. Salvetti E. Franz C. M. A. P. Harris H. M. B. Mattarelli P.. (2020). A taxonomic note on the genus *Lactobacillus*: description of 23 novel genera, emended description of the genus *Lactobacillus* Beijerinck 1901, and union of *Lactobacillaceae* and *Leuconostocaceae*. Int. J. Syst. Evol. Microbiol. 70, 2782–2858. doi: 10.1099/ijsem.0.004107, PMID: 32293557

