# Supplementary figures and images for: Glycerol-derived reuterin regulates human intestinal microbiota and metabolites
Source: Front Microbiol. 2024 Oct 18;15:1454408. doi: 10.3389/fmicb.2024.1454408 (PMC11527728; doi:10.3389/fmicb.2024.1454408)

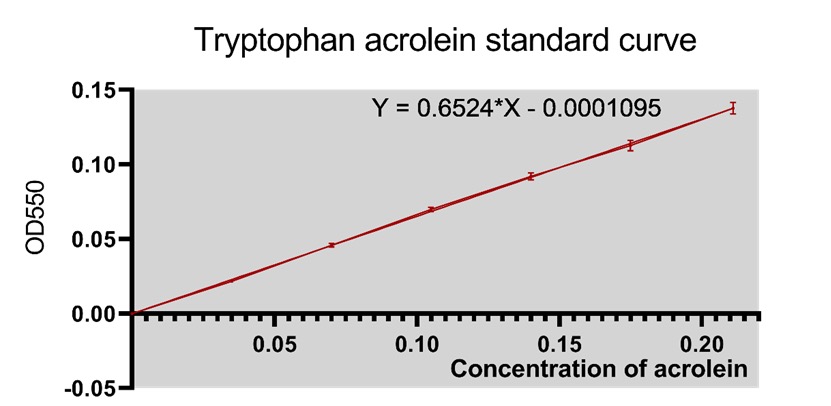

Supplement: SUPPLEMENTARY FIGURE S1 — Tryptophan acrolein standard curve. The X-axis is the concentration of acrolein, in mM, and the Y-axis is the OD550 value. Each point represents the mean ± SD of triplicate determinations. [file Image_1.JPEG]

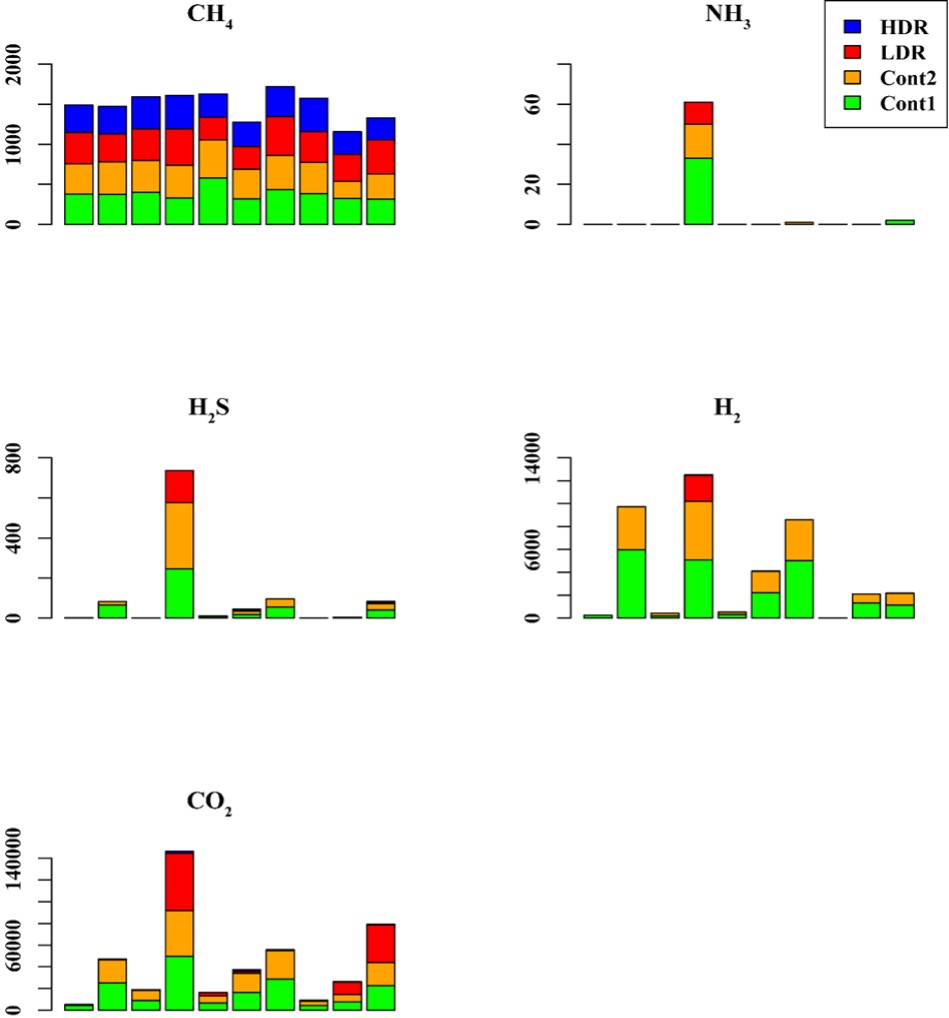

Supplement: SUPPLEMENTARY FIGURE S2 — Gases produced by each candidate after fermentation for 24 h in vitro. The X-axis represents the order of candidates, and the Y-axis represents the corresponding amount of gas produced. The green, orange, red, and blue colors represent the Control 1, Control 2, low-dose reuterin, and high-dose reuterin groups, respectively. [file Image_2.JPEG]

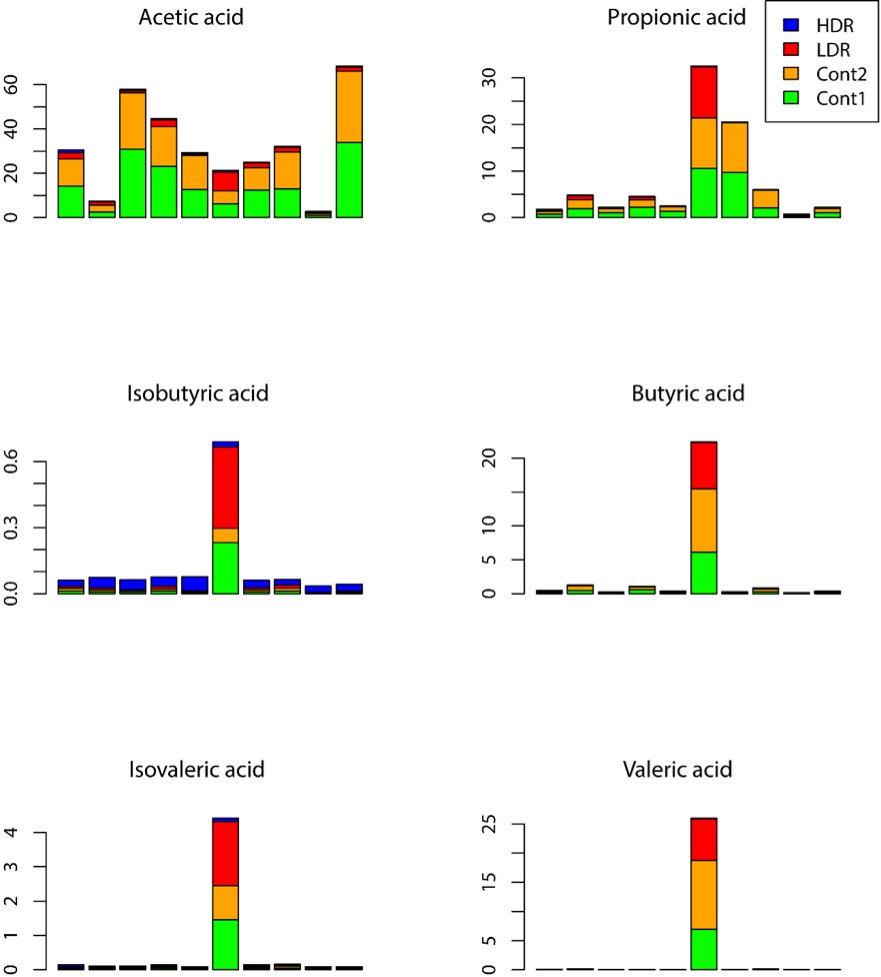

Supplement: SUPPLEMENTARY FIGURE S3 — SCFAs produced by each candidate after fermentation for 24 h in vitro. The X-axis represents the order of candidates, and the Y-axis represents the relative amounts of SCFAs produced. The green, orange, red, and blue represent the Control 1, Control 2, low-dose reuterin, and high-dose reuterin groups, respectively. [file Image_3.JPEG]

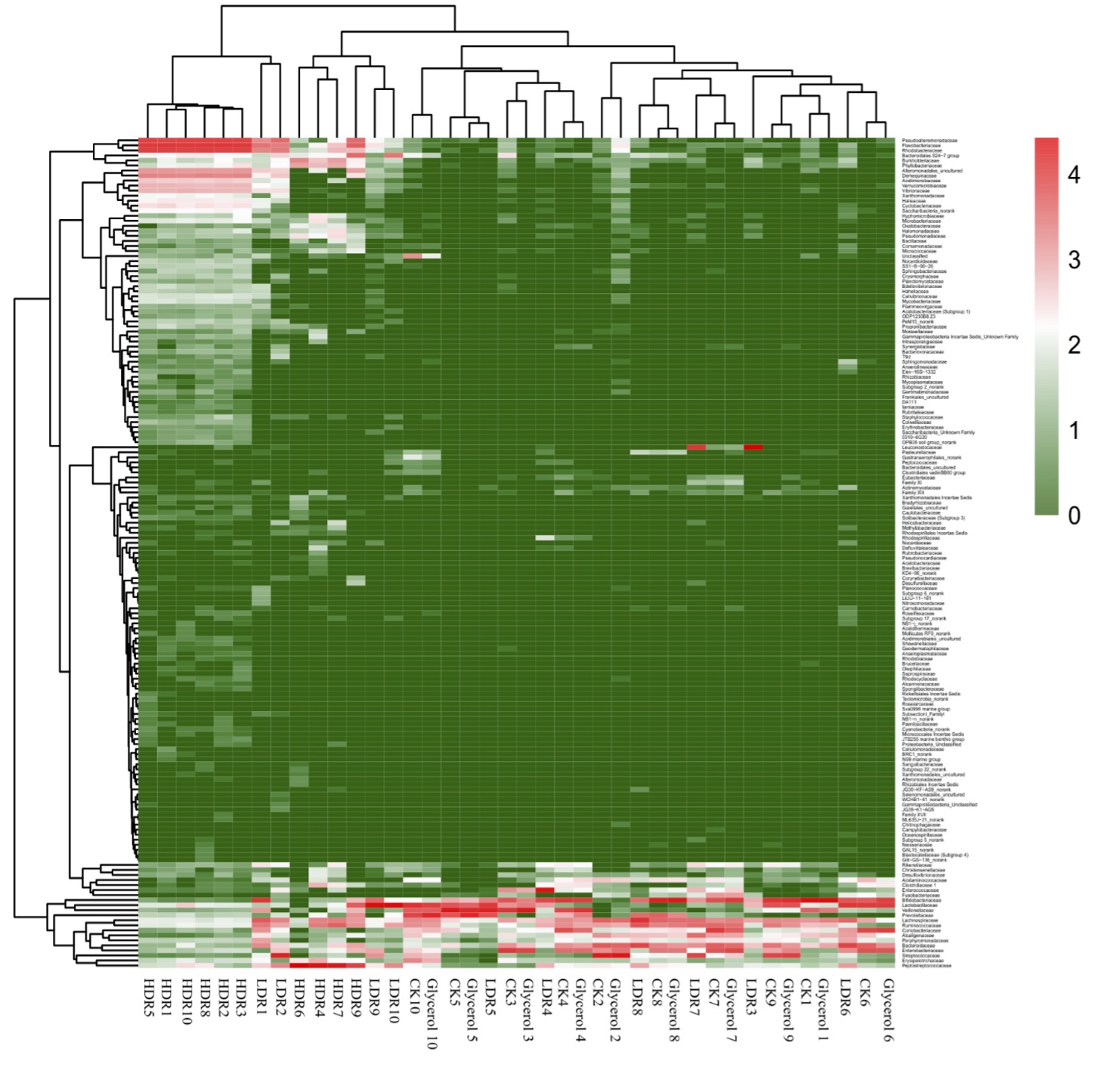

Supplement: SUPPLEMENTARY FIGURE S4 — Absolute bacterial abundance at the family level in each candidate after fermentation for 24 h in vitro. The X-axis represents the order of candidates in each group. The left Y-axis represents the cluster of bacterial composition in each group, and the right Y-axis represents the family names of bacteria. [file Image_4.JPEG]

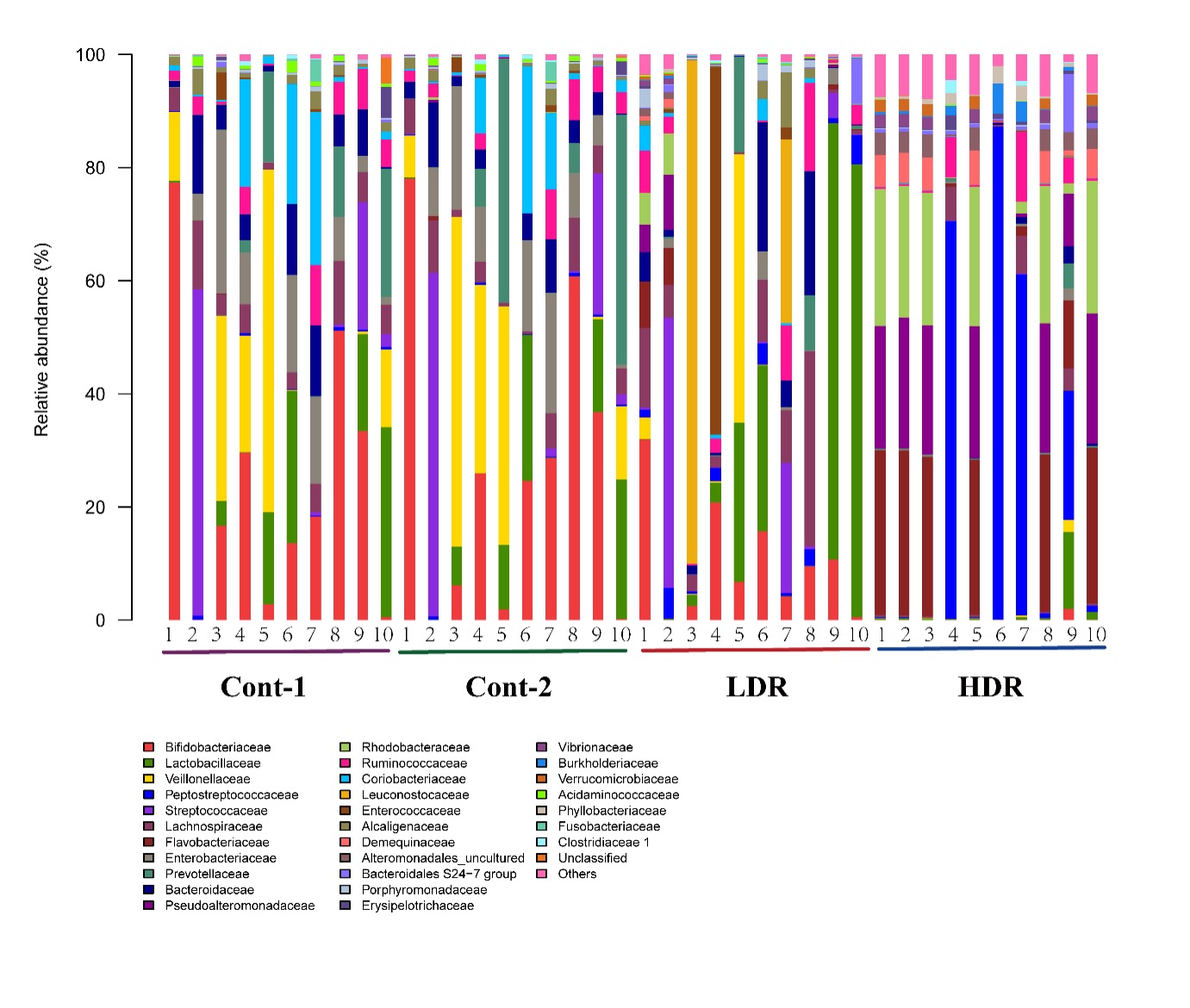

Supplement: SUPPLEMENTARY FIGURE S5 — Relative abundances of the bacterial community at the family level. Each bar shows the distribution of species in each sample. The number on the X-axis represents the sample number. The Y-axis shows the relative abundance of bacteria at the family level in each sample as a percentage. The purple, green, red, and blue lines under each sample represent the sample groups for each culture condition: YCFA medium only (Cont-1), YCFA medium plus glycerol (Cont-2), and FCFA medium with low (LDR) and high (HDR) reuterin concentrations. [file Image_5.JPEG]
